# Predicting fixations and gaze location from EEG

**DOI:** 10.1007/s11517-025-03362-6

**Published:** 2025-05-08

**Authors:** Yoelvis Moreno-Alcayde, V. Javier Traver, Luis A. Leiva

**Affiliations:** 1 Institute of New Imaging Technologies, Universitat Jaume I, Av. Vicent Sos Baynat, s/n, 12071 Castellón, Spain; 2 University of Luxembourg, 6, avenue de la Fonte, L-4364 Esch-sur-Alzette, Luxembourg

**Keywords:** EEG, Eye-gaze, Fixation, Neural models

## Abstract

**Abstract:**

Brain signals carry cognitive information that can be relevant in downstream tasks, but what about eye-gaze? Although this can be estimated with eye-trackers, it can be very convenient in practice to do it without extra equipment. We consider the challenging tasks of fixation prediction and gaze estimation from electroencephalography (EEG) using deep learning models. We argue that there are three critical design criteria when designing neural architectures for EEG: (1) the spatial and temporal dimensions of the data, (2) the local vs global nature of the data processing, and (3) the overall structure and order with which the steps (1) and (2) are orchestrated. We propose two model architectures, based on Transformers and LSTMs, with different variants in this large design space, and compare them with recent state-of-the-art (SOTA) approaches under two constraints: reduced EEG signal length and reduced set of EEG channels. Our Transformer-based model outperforms the LSTM-only model, but it turns out to be more sensitive with short signal lengths and with less number of channels. Interestingly, our results are similar or slightly better than SOTA, and the models are trained from scratch (i.e., without pre-training or fine-tuning). Our findings provide useful insights for advancing in eye-from-EEG tasks.

**Graphical abstract:**

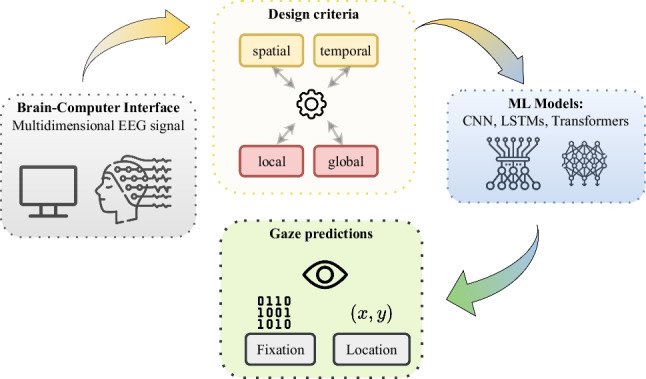

## Introduction

Brain-computer interfacing (BCI) allows to decode relevant cognitive internal states such as affect or cognitive load [2, 50], although when it comes to decoding eye-related data, such as fixations and gaze location, current BCI-based methods are still behind eye-trackers [17, 40]. Eye-trackers allow to accurately estimate human visual attention, which is relevant to many interactive tasks [3]. However, most eye-trackers are video-based, and therefore, they require a direct line of sight from the eyes to the camera. Wearable eye-trackers could be an alternative, but they have limited accuracy [43].

Researchers have explored the benefit of the joint use of gaze and brain signals [1, 16, 22, 26, 51] or relate gaze patterns with brain activations [44]. If we could reliably estimate eye-related data from brain signals, this would remove the need for setting up and calibrate two devices. Also, this would be the only alternative for people with locked-in syndrome, who have extremely limited eye movements [39]. The cortical areas involved in eye movements were studied by Grosbras et al. [9]. A BCI-based visual speller without eye movements was demonstrated by Treder et al. [42]. In this paper, we contribute a modelling study that analyses spatio-temporal representations of brain signals (as captured by electroencephalography, or EEG) in two particular tasks: predicting gaze fixations and estimating gaze coordinates.

Previous work has investigated the problem of estimating eye activity from brain data [10, 40]. EEGNet [25] was compared with a shrinkage linear discriminant analysis for the problem of fixation classification, with improved performance [21]. Deep learning models for time-series segmentation of the EEG signal have been explored for eye events detection [48]. Linear models have been explored for blink and saccade detection using electrooculography data as ground-truth [6].

Currently, there are few public datasets that include both EEG and eye data, but only one that can be used for the purpose of this work. For example, the *Zurich Cognitive Language Processing Corpus (ZuCo)* dataset [12] provides recording of people reading natural English sentences, aimed at investigating cognitive processes behind human reading and language understanding. The nature of the reading task, consisting mostly of microsaccadic and mostly horizontal eye movements, is not general enough. Shafiei et al. [38] collected a dataset for the analysis of robot-assisted surgery. Pei et al. [35] released a dataset to compare the visual concentration of athletes with that of non-athletes; although related, the task is not suitable for our study (e.g., there is a single fixation point per trial) and the dataset is rather small for deep learning (DL) models. In the context of neuromarketing, the NeuMa dataset [8] includes brain, eye, and mouse data of participants while browsing supermarket brochures who were asked to select which products they would buy. The variety of visual stimuli in the brochures is likely to involve other cognitive processes that could interfere with regular eye movements. The amount of data is also limited. Finally, the EEGEyeNet dataset includes synchronised eye and EEG data in target acquisition tasks [17]. Although the tasks were performed in controlled lab conditions, this dataset is general enough for our research purpose and large enough for DL models. Also, there is previous work that has used this dataset [7, 47, 49]; therefore, we can compare and contrast our models against the state of the art (SOTA). Although Riemannian geometry-based models have some potential for EEG processing for BCIs [41], they were not found more effective than other machine learning models in EEG-based eye-related classification and regression tasks [52]. For saccade direction classification, feature selection for traditional classifiers was separately applied to the first and second temporal halves of the EEG signals of all participants [31], resulting in smaller training times and generally better performance.

Yang et al. [49] used a Vision Transformer (ViT) architecture to predict gaze positions from EEG signals. Interestingly, they reported improved performance if the ViT was pretrained on ImageNet [5], a large-scale general-purpose dataset of natural images. Building on that ViT, recent work has explored the effect of kernel sizes [36] and proposed to include clustering-based data pre-processing and separable convolutions [19]. Seeking smaller and faster models, the lightweight MobileViT model [30] has been used, along with knowledge distillation, for EEG-based gaze prediction [28]. Another approach [7] used a deep convolutional neural network (CNN) inspired by ResNet [11], one of the most popular CNN models. Self-attention layers have been included in CNNs for EEG classification tasks [46]. Similarly, attentional blocks have been proposed [47] for increasing interpretability, by analysing attention weights to find correlations between their inputs. The benefit of including an EEG reconstruction module was studied by Li et al. [27]. Recently ViTs are combined with temporal convolutional networks [32], which were previously studied in motor-imagery BCI tasks [14].

The contributions of this work are as follows:

- A conceptual framework to discuss previously proposed models and motivate new ones, in terms of how the spatio-temporal and the local-vs-global patterns of the EEG signal are processed.
- Two new deep learning models for *EEG-based eye-tracking* combining convolutional neural networks, long short-term memory cells, and encoder Transformers, conceived by discussing the proposed framework. Our models are tested on two eye-related problems: fixation prediction (binary classification) and gaze estimation (regression task).
- An analysis of the performance of the proposed models under two constraining conditions: having fewer EEG channels and temporally shorter signals.
- Comparison of existing models and our novel proposals, in terms of (a) their architectures under the insights of the proposed framework and (b) experimental performance in gaze prediction, obtaining results which are on par or better than state of the art.

**Fig. 1:**
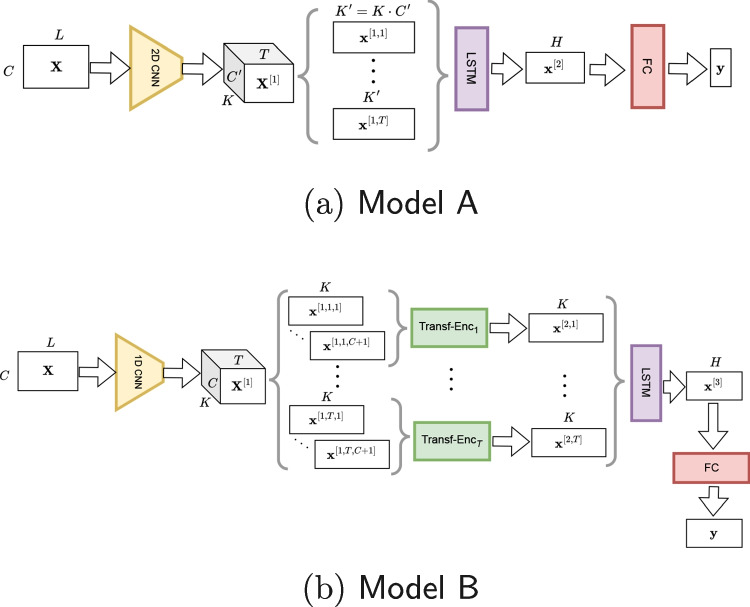
Structure of **a** Model A and **b** Model B. The CNNs are fully convolutional (i.e. without dense layers). In Model A, the CNN is 2D rather than 1D, and there are no intermediate Transformers

## Method

### Spatio-temporal representations

EEG signals are time series derived from a number of EEG channels placed on the scalp with a particular spatial configuration, usually following the standardised 10-20 system [20]. Therefore, there are two key dimensions to take into account to analyse these signals: the spatial relationship among the different EEG electrodes and the temporal evolution (dynamics) of the EEG signal. The frequency domain might be another dimension to consider, but our study focuses on the spatio-temporal domain, as in the closest related work, and the analysis of frequency domain is left for possible future work. At the same time, in addition to this spatio-temporal dimension, it is important to consider how the EEG information is processed, either locally or globally, in the spatial, temporal, or spatio-temporal domains. Finally, how are the processing modules connected may have an impact on the learned representations and their discriminative power. The order and structure of these modules usually relate to the semantic level of the data they deal with, from the low-level input data to the higher-level feature vectors and learned embeddings.

Clearly, these dimensions (or design elements) entail high degrees of freedom, and also a large design space where it is not obvious which approach can be more appropriate. For example, should the input signal be split into temporal windows and processed independently, or proceed with a fully global encoding first? Similarly, it is not clear how the different EEG channels may be processed more conveniently. Beyond being a design reasoning aid, this design space can be useful for comparing different approaches and may help in providing insights into their relative merits or even some basis for explaining their respective performances. An additional benefit of using this design space is to help thinking in terms of high-level and problem-related concepts, instead of directly focusing on known model architectures. As a matter of fact, different models can be used and combined in multiple ways, but investigating the underlying principles can be more important than the fact that a specific known architecture is used. In this paper, we propose two DL models and compare them against three SOTA models.

### Our first approach: Model A (2D-Ta)

A diagrammatic representation of this model is given in Fig. 1a. Our input data is $\textbf{X}$, the preprocessed EEG signal for all *C* channels each of a given length *L* (number of temporal samples). We use superscripts [*k*] to indicate the processing step *k* and, where required, [*k*, *l*] or [*k*, *l*, *m*], to represent slices of the tensor within the same processing step *k*.

We first perform a **local spatio-temporal** processing so that we get embeddings that account for the neighbouring embedding in both directions of the 2D array $\textbf{X}$. A two-dimensional CNN model is suitable for this purpose:

1 $$\begin{aligned} \textbf{X}^{[1]} = \textsf {CNN-2D(\textbf{X} )}, \qquad \textbf{X}^{[1]} \in \mathbb {R}^{K \times C'\times T} \end{aligned}$$

The resulting three-dimensional volume $\textbf{X}^{[1]}$ has *K* channels, corresponding to the multiple convolutional filters in the internal CNN layers, and $C'$ and *T* corresponds to the reduced sizes (after convolutions and pool operations) of the input $C\times L$ array, so $C'<C$ and $T<L$.

Now, this volume is reshaped into a sequence of *T* embedding vectors, and we capture the temporal relationships between *T* outputs (each of size $K' = K\cdot C'$) to have a summarised representation. This can be understood as a mixture of local and global processing steps. It is temporally local in the sense that input vectors are processed sequentially, but it is global because a summarised output (context) is produced by considering the whole sequence. Thus, we can refer to this processing as **temporally “glocal”** (global + local), for which an LSTM model is suitable:

2 $$\begin{aligned} \textbf{x}^{[2]} = \textsf {LSTM}\left( \left\{ \textbf{x}^{[1,t]}\right\} _{t=1}^{T}\right) , \qquad \textbf{x}^{[2]} \in \mathbb {R}^{H\times 1} \end{aligned}$$

where *H* is the size of the hidden vector representation.

Finally, a fully connected block (dense layers) provides a **global spatio-temporal** prediction:

3 $$\begin{aligned} \textbf{y}  = \textsf {FC}(\textbf{x}^{[2]}). \end{aligned}$$

We also considered a variation of Model A with one Transformer that replaces the LSTM, which we denote it as Model A $^\text {tt}$ (“tt” standing for “temporal transformer”), which achieved slightly lower performance. A possible explanation of why the LSTM-based approach outperformed the Transformer-based one is that the temporal sequence in this case is not very long and the LSTM can successfully capture the required temporal information. Another reason is that while Transformers are more flexible and general, more training data is required to exploit that flexibility.

After that, two further motivations for model improvement were as follows. On the one hand, it could possibly be better to perform only 1D (temporal) convolutions, not 2D (spatio-temporal), since neighbouring channels in the input data matrix do not properly capture the spatial layout of the EEG electrodes. On the other hand, the Transformer model, which processed data globally in the temporal domain, was replaced by a Transformer to operate in the spatial domain, also globally. We refer to this as Model A $^\text {st}$ after “spatial Transformer.” It turned out that the spatially focused Transformer outperformed the temporally focused Transformer. This observation confirms our intuition that capturing the spatial relationships among the channel representations is beneficial, and better than using 2D convolutions. An additional hypothesis was that the temporal processing could be done locally rather than globally, guided by the results with the LSTM in Model A. Therefore, instead of a single spatially focused Transformer, we could have *multiple* Transformer models: one per temporal segment. In this way, both the temporal and the spatial dimensions were captured in a richer manner. This led to our next proposal, Model B, described in the next section.

### Our second approach: Model B (1D-Sa-Ta)

A diagrammatic representation of this model is given in Fig. 1b. The input data is the same as in Model A, $\textbf{X}$. The processing has two main differences. First, the local spatio-temporal (hence, 2D) processing is replaced by a local (*only*) temporal, since the spatial (channel-wise) relationships between channels in Model A relies on a limited and arbitrary relationship of their order in the 2D array, and we argue this is not an ideal modelling decision. Second, the output of this first step does not go directly to the temporal processing, but it is previously processed to capture the spatial (channel) relationships more globally and comprehensively, yet retaining the different temporally local characteristics.

Therefore, the first processing is **local temporal** where each channel is treated independently so that we get a temporal encoding both segment-wise and channel-wise. For that purpose, conventional 2D CNN models can be used to process all the channels simultaneously, even though they are actually processed separately (i.e. through 1D convolutions):

4 $$\begin{aligned} \textbf{X}^{[1]} = \textsf {CNN-1D}(\textbf{X} ), \qquad \textbf{X}^{[1]} \in \mathbb {R}^{C\times K\times T} \end{aligned}$$

where the output $\textbf{X}^{[1]}$ has lower temporal length ($T<L$) due to the convolutions and pooling layers of the CNN-1D, but the number of channels (*C*) is kept. In addition, due to the convolutional and pooling layers, we have a 3D volume ($C\times K\times T$) with *K* activation outputs per channel and temporal step. We call this a *local* processing because the convolution filters and the pooling operations are local in nature.

Next, we want to capture the **global spatial** relationships between channels and do it **locally** per temporal segment. To that end, $\textbf{X}^{[1]}$ is split into *T* groups of sequences each of length *C*, and each sequence is formed by embedding vectors of length *K*, $\textbf{x}^{[1,c,t]}, c\in [1,C], t\in [1,T]$, with *c* indexing channels within each sequence, and *t* indexing each of the *T* sequences. In order to capture the relationships between channels, we use a Transformer encoder. Since Transformers include pairwise-relationships, all possible channels representations are compared, and hence, this is considered global spatial processing. Note that the input sequence to each Transformer encoder is not a temporal sequence (as words in text or frames in video processing, for example), but a spatial one. An alternative model to capture the channel representation would be a graph neural network (GNN), but this is left to future work.^1^ The output for each of these *T* encoders is a *K*-dimensional vector

5 $$\begin{aligned}&\textbf{x}^{[2,t]} = \textsf {Transf-Enc}_t \left( \left\{ \textbf{x}^{[1,c,t]}\right\} _{c=1}^{C+1}\right) , \quad t\in [1,T], \nonumber \\  &\textbf{x}^{[2,t]} \in \mathbb {R}^{K\times 1} \end{aligned}$$

Then, a **temporally “glocal”** processing is performed as in Model A, with an LSTM and FC block being used as well:

6 $$\begin{aligned} \textbf{x}^{[3]} = \textsf {LSTM} \left( \left\{ \textbf{x}^{[2,t]} \right\} _{t=1}^{T} \right) , \qquad \textbf{x}^{[2,t]} \in \mathbb {R}^{K\times 1} \end{aligned}$$

7 $$\begin{aligned} \textbf{y}  = \textsf {FC}(\textbf{x}^{[3]}). \end{aligned}$$

Note that Model A and Model B are both end-to-end architectures including a 2D CNN (Model A) or a 1D CNN (Model B), followed by a set of Transformers (encoder part) only in the case of Model B, and final LSTM and FC blocks. The details for all these parts and their training are given in Appendix 6. The names for both models, 2D-Ta, and 1D-Sa-Ta, compactly capture their respective main traits: “2D” and “1D” denote the processing of the 2D input data array (channels $\times$ time); while “Sa” and “Ta” represent their subsequent “space aware” (Sa) and “time aware” (Ta) parts.

### Eye-related tasks

Two relevant tasks are considered: fixation event prediction and gaze location prediction. The analysis of fixation events (whether a user is fixating or not for some time) is important since it reveals attentional spans in user interactions [37]. Estimating the gaze coordinates on screen is also important and very relevant for the analysis of visual salience and scanpath patterns [4]. We should note that, in this case, the ground-truth coordinates are the gaze points from the eye tracker, not the actual on-screen target point. Therefore, for the same target point, different ground-truth points exist per participant.

### Signal cropping

Although the analysis of different spatio-temporal representations is the main objective of this work, we also consider the impact on performance of the length of the brain signal. Given how the dataset is prepared, where the fixation is mostly centred, we expect the most discriminative information to be in the centre of the signal, and we want to assess how much signal or context is actually required.

To study this effect, a ratio $\gamma$ the central part of the signal is kept while removing a ratio $\rho =\frac{1-\gamma }{2}$ to the left and to the right of the signal. The performance is evaluated with increasing cropping ratio $\rho \in \{0,0.1,0.2,0.3\}$, which equals to keeping decreasing signal ratios $\gamma \in \{1,0.8,0.6,0.4\}$.

### Impact of using fewer EEG channels

Another practical aspect of our interest is to evaluate the impact of the number of channels in task performance. This impact has been addressed in the past for a variety of applications, such as brain imaging [24], tracking speech [33], or epileptic source localization [23]. The results suggest that the number of required channels can be reduced without a significant drop in performance [13, 24, 33]. Some works have demonstrated that single-electrode devices may suffice for certain tasks such as wheelchair control [34]. Through gradient-based method, the most important electrodes can be identified, with the frontal ones being more discriminating for eye movement, and 23 out of 128 electrodes can be equally predictive [18]. For high precision tasks, however, denser electrode distribution are required [23].

**Table 1:** Dimensions of input data and intermediate matrices processed by the proposed models

	Model A and Model B					Model A	
No. channels	*L*	*C*	*K*	*T*	*H*	$C'$	$K'$
129	500	129	128	29	768	6	768
8	500	8	128	29	768	8	1024

Our particular goal was to compare medical-level BCI devices such as the high-density 128-channel system used for EEGEyeNet [17] to consumer-level systems such as the sparser Unicorn Hybrid black^2^ with only 8 channels (7 plus a reference one). We therefore selected the corresponding channels {Fz, Cz, C3, C4, Pz, OZ, PO7, PO8} for comparison. Notice that having fewer electrodes is just one characteristic of the consumer-grade BCI devices, and therefore, this is only an approximation.

## Experiments

### Dataset and setup

We used the previously discussed EEGEyeNet dataset [17]. It features EEG and eye-tracking recordings from 356 participants. Data from three tasks are included: left-right, angle/amplitude, and absolute position. Each data point corresponds to 1 s signal at 500 Hz, i.e., $L=500$ time steps for $C=129$ electrodes (128 plus a reference one). The dataset includes data preprocessed at two levels: minimally and maximally processed data. Minimally processing detects and interpolates bad channels, and band-pass filter the data in the [0.5, 40] Hz. Maximally processing, additionally, removes artefacts, including eye ones. Since it is known that maximally processing lowers the performance [18], we focus on minimally processing. As stated above, we consider two tasks for our experiments: gaze fixation prediction and gaze location prediction.

#### Fixation event prediction

Since the original dataset [17] does not explicitly consider any task for fixation analysis, we prepared the data for this binary classification task by combining data from two other tasks: the $N_\text {fix}=17,830$ positive examples correspond to the data in the angle/amplitude task, and the $N_{\text {no fix}}=21,464$ negative examples were taken from the absolute position task. The training, validation, and test sets correspond to the union of the corresponding subsets as originally proposed [17, Table 3].

#### Gaze location prediction

This task corresponds directly with the absolute position task, and it is formulated as a regression problem. The total number of data points in the dataset is 21, 464.

In both tasks, 70/15/15 (%) splits are used for training, validation, and testing, respectively.

The dimensions of the matrices involved in the processing steps of both models are given in Table 1 for the case of no-cropping and using either all the electrodes or the reduced channel set.

### Compared models

Our models are compared with three proposals (EEGViT, DCNN, and Attention-CNN), which are described here in terms of the framework used to motivate our models above.

#### Hybrid vision Transformer (EEGViT) [49]

The EEGViT architecture [49] features a convolutional part followed by a Visual Transformer (ViT). The input to the convolutional part is a 2D array $\textbf{X}$ representing the signal of the *C* EEG channels along *T* time units. The convolutional part includes a first 1D convolutional layer filtering along time (**local temporal** processing) with $1\times t$ filters followed by a second 1D convolutional layer filtering along channels with $c\times 1$ filters. This convolutional part is assumed to be equivalent to process the input 2D array into separated $c\times t$ patches. The encodings corresponding to each of these patches are the input tokens to the ViT, which can therefore be understood as a **global spatio-temporal** processing.

#### DCNN [7]

A single deep CNN is proposed by Fuhl et al. [7], with the following aspects. First, 16 linear combinations of all channels is applied at each time step using per-channel learnable $1\times 1$ convolutions (i.e. $C\times 1$ filters). This corresponds to **globally spatial** (because of the linear combination, where no spatial relationship is assumed) and **temporally local** (because this is computed per time step) processing. Two residual blocks include convolutions and pooling along time, but including all channels, thus representing **temporal and local** and **spatial global** processing. The two final fully connected layers represent **global spatio-temporal** representations.

#### Attention-CNN [47]

Weng et al. [47] proposed a CNN to process EEG signals through convolutions only in the temporal domain (**temporally local** processing), but the CNN features also attentional blocks to learn to weight the importance of individual EEG channels for the target task. Two types of attentional blocks are used: squeeze and excitation (SE) and self-attention (SA). The squeeze operation summarises the 2D (channel $\times$ time) activation maps into 1D (channel-only time), which can be understood as another form of temporally local operation. The excitation operation combines the information of the channels; thus, it is a **spatially global** process. These SE blocks are optionally applied sequentially after each of the 4 residual blocks. Then, a SA block is intended to relate the different channels, i.e. another **spatially global** operation. A final fully connected layer would roughly correspond to a **spatio-temporally global** procedure.

#### Comparison of models

The idea of performing temporal-only convolutions is a shared design aspect in our Model B and also in EEGViT and Attention-CNN. This reflects the concern of filtering the 2D channel $\times$ time input array with 2D filters since this assumes a spatial relationship that is not actually true, and might affect the subsequent representation. However, EEGViT subsequently performs additional spatial-only convolutions, and our Model A performs 2D convolutions. Also, the CNN parts in our models and in EEGViT are fully convolutional, in contrast to Attention-CNN and DCNN which includes fully connected layers.

The temporal processing is more explicitly present in our models through the LSTMs. The EEGViT somehow performs simultaneous spatio-temporal processing through the ViTs applied to local spatio-temporal patches. Interestingly, our Model B includes two levels of temporal processing, first more locally as part of each of the Transformer encoders which focus on a specific temporal segments, and then more globally through the LSTM whose input sequence encode richer semantic information. The temporal characterisation of Model B through this 2-level hierarchical processing is potentially richer than the other existing alternatives.

The spatial relationships among channels are captured through different approaches. In our Model A, they are more limited and local through the CNN, whereas in Model B they are heavily exploited through the temporally focused Transformer encoders. In Attention-CNN, attentional blocks are in charge of capturing this relationship.

### Performance assessment

For the fixation event prediction task, we used classification accuracy, to better compare against previous work. We note that both classes are mostly balanced ($45\%$ and $55\%$ of instances per class). For the gaze location prediction task, the root mean square error (RMSE) and the mean absolute error (MAE) are used, following previous work [17, 18, 47, 49].

Let $\textbf{y}  = (x_i,y_i)$ be the ground-truth gaze point and $\hat{\textbf{y} } = (\hat{x}_i,\hat{y}_i)$ the predicted gaze point from the EEG data, then for the *n* test data points, the metrics are computed as

8 $$\begin{aligned} \text {RMSE} = \sqrt{\text {MSE}},\quad \text {MSE} = \frac{1}{2n}\sum _{i=1}^n (x_i - \hat{x}_i)^2 + (y_i - \hat{y}_i)^2, \end{aligned}$$

9 $$\begin{aligned} \text {MAE} = \frac{1}{2n}\sum _{i=1}^n |x_i - \hat{x}_i| + |y_i - \hat{y}_i|. \end{aligned}$$

For performance evaluation, all models are trained 5 times with different weight initializations, and the corresponding mean and standard deviation of performance metrics on the test set are reported.

**Fig. 2:**
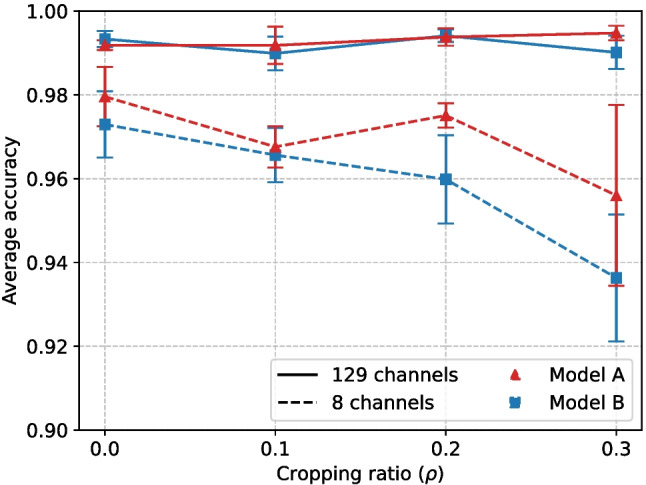
Fixation prediction. Influence of the number of channels and signal cropping for Model A and Model B

### Results

### Fixation prediction

When using all EEG channels, both Model A and Model B achieve very good performance in estimating fixation events (Fig. 2), with an average accuracy around 99%. We also inspected the area under the ROC curve (AUC), and the difference between both models was even smaller. This high performance suggests that fixation event prediction from EEG signals is a relatively easy task. Under the reduced EEG channel set, performance slightly drops, but it is still very high (about 95% average accuracy).

In this case, Model A outperforms Model B, mostly for higher cropping ratios $\rho$. A possible interpretation is that the additional complexity of Model B does not pay off in this simple task, particularly with shorter segments corresponding to higher cropping ratios $\rho$. Compared with the full 129-channel set, where the performance is virtually unaffected by cropping, both models suffer from signal cropping in the 8-channel case. This suggests that the redundancy associated with more channels helps to compensate the lack of discriminative information which is lost due to signal cropping. Interestingly, in both cases, performance is largely unaffected by the cropping rate, possibly because the most discriminative information is in the centre of the signal segment, which is mostly retained even with the larger cropping ratios $\rho$.

**Table 2:** Performance as $mean{\scriptscriptstyle \pm std. dev.}$ (in mm) on **gaze estimation** with different approaches. $\downarrow$ means “lower is better”

	CNN	Attention-CNN	EEGViT	DCNN	Model A	Model B
	[17]$^{a}$	[47]	[49]	[7]	(ours)	(ours)
RMSE $$\downarrow $$	$$70.2{\scriptscriptstyle \pm 1.1}$$	$$54.79{\scriptscriptstyle \pm 0.12}$$$$^{b}$$	$$55.4{\scriptscriptstyle \pm 0.2}$$	N/A	$$55.68{\scriptscriptstyle \pm 0.27}$$	$$54.85{\scriptscriptstyle \pm 1.13}$$
MAE $\downarrow$	N/A	N/A	$39.64{\scriptscriptstyle \pm 0.85}$	$49.20{\scriptscriptstyle \pm 0.60}$	$40.11{\scriptscriptstyle \pm 1.17}$	$38.88{\scriptscriptstyle \pm 0.78}$

$^{a}$Best-performing model tested on dataset introduction

$^{b}$Not directly comparable since an extended dataset is considered

**Fig. 3:**
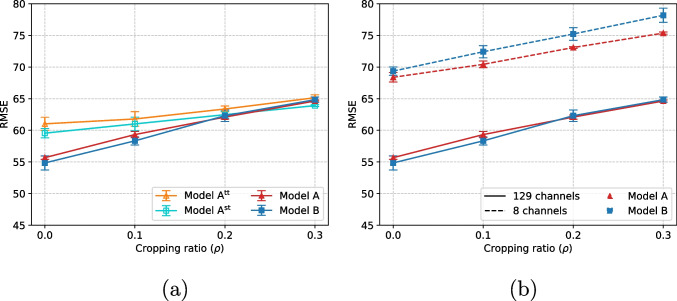
Model comparison (RMSE: lower is better) for increasing cropping rates (horizontal axis), with **a** full channel set and **b** full vs reduced channel set

### Gaze location prediction

We first analyse our models under the best-case conditions used by compared methods, i.e. using all channels, minimally processed signals, and with no cropping. The results (Table 2) indicate that Model B performs (slightly) better than Model A, and that both models are competitive, and Model B offers marginally better performance than recent approaches. As stated in the table, the result reported by Fuhl et al. [7] uses a different version of the dataset, and therefore, it is not directly comparable to our results; arguably, our respective performances are similar.

We now compare Model A and Model B under varying cropping ratios and two channels sets. It can be observed that when using all the channels (Fig. 3a), Model B slightly outperforms Model A, and that both Model A and Model B significantly outperform the tentative models Model A $^\text {tt}$ and Model A $^\text {st}$. This highlights that the design ideas behind Model B were sound and turned out to be effective. For the reduced channel set, the performance decreases for both models (Fig. 3b), as expected, but the behaviour is reversed, and Model A outperforms Model B. As in the fixation case, this gap increases with the cropping rate $\rho$, which suggests that Model B is more sensitive than Model A to shorter signal segments.

**Fig. 4:**
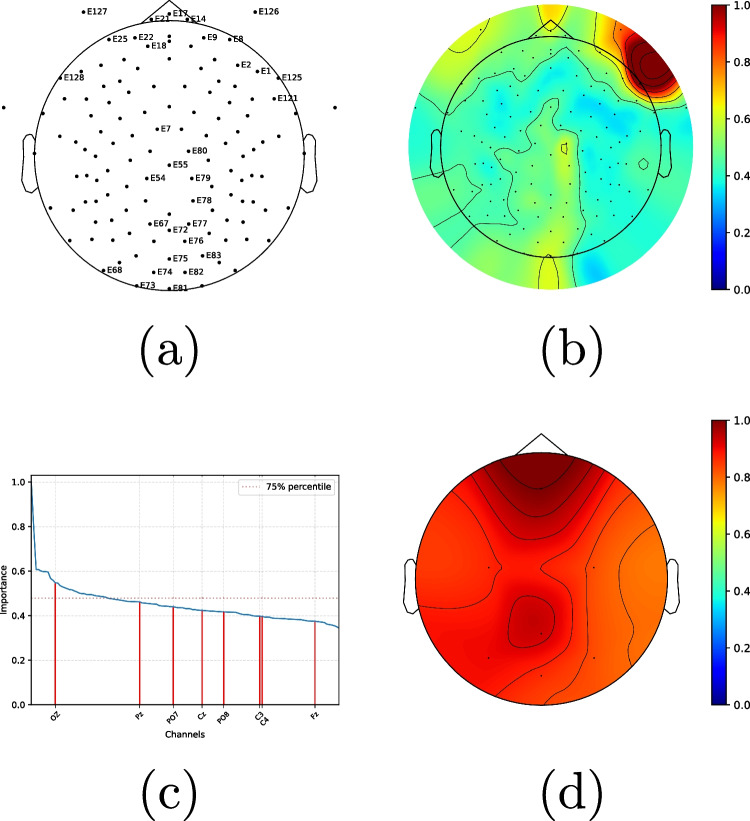
Attention visualisation: **a** top 25% channels (32 out of 128); **b** importance map computed from all channels (128); **c** plot of decreasing importance for all 128 channels, with the 8 electrodes of the 8-channel case being highlighted; and **d** importance map computed from the set of 8 channels

### Visualisation of channel importance

We now visualise the relevance of the channels for prediction based on the self-attention mechanism in the Transformer encoders used in Model B.

To that end, for each test instance *i*, we consider the self-attention weight matrix $\textbf{A}^i_t$ of the last layer for each of the *T* Transformer encoders ($\textsf {Transf-Enc}_t, t\in [1,T]$):

10 $$\begin{aligned} \textbf{A}^{(i)}_t = \textsf {SoftMax}\left( \frac{\textbf{Q}_i^T\cdot \textbf{K}_i}{\sqrt{d}}\right) , \end{aligned}$$

where $Q_i$ and $K_i$ are the query and key matrices for the *i*-th test data point.

We keep the first row of this matrix corresponding to the CLS channel, since it is the one used at the output (Fig. 1) and the relevant one for the prediction. This row represents the attention received from the rest of the channels $\textbf{a}^{(i)}_t= \mathbf [{A}_t]_{1,:}$, which are averaged over the *T* vectors to get the importance vector

11 $$\begin{aligned} \varvec{\alpha }^{(i)} = \frac{1}{T} \sum _{t=1}^T \textbf{a}^{(i)}_t \end{aligned}$$

for the *i*-th instance. Finally, the channel-wise importance is averaged over the *m* test instances:

12 $$\begin{aligned} \varvec{\alpha } = \frac{1}{m} \sum _{i=1}^m \varvec{\alpha }^{(i)} = [\alpha _1,\ldots ,\alpha _{128}], \end{aligned}$$

where each $\alpha _i \in [0,1]$ is assumed to represent the “importance” of the *i*-th channel.

The results (Fig. 4) suggest that many of the selected channels and the importance distribution correspond to frontal electrodes, in agreement with existing evidence [47].

For the 8-channel subset, the higher importance is also localised in the frontal part (and more centred than in the 129-channel case), although the difference between importance at different regions is weaker. A possible interpretation for this more uniform importance distribution is a compensation mechanism against the sparser electrode placement.

## Discussion

Our results pertaining the gaze location tasks are similar or slightly better than SOTA results, without any pre-training as required in a recent ViT-based approach [49]. Compared to Transformers, our LSTM-based model accounting for temporal EEG dynamics is found competitively powerful. Interestingly, additionally learning spatial (channel-wise) representations prior to the temporal modelling is found to be beneficial. This was implemented as a set of Transformer encoders, each focusing on non-overlapping time windows. By leveraging the self-attention mechanism, the input to each of these encoders is not an actual (temporal) sequence, but separate per-channel feature vectors.

It was observed that using significantly fewer EEG channels has no dramatic effect on the fixation prediction task, but notably more impact on the harder gaze estimation task.

Although the performance is not severely affected by the amount of cropping, the full set of EEG channels fares better the cropping, arguably because the lack of signal is smartly compensated with the richer (albeit possibly somehow redundant) information from more channels.

The proposed Transformer-based model seems to capture the spatio-temporal relationships, and it can be more effective than the simpler LSTM-based model, but it tends to be less robust to the length of the signal when using fewer channels, as found with increasing cropping ratio. An explanation is that, on the one hand, with fewer channels, each Transformer has shorter sequences as input, and the Transformer’s ability to capture spatial relationships weakens because of the fewer electrodes and their sparser distribution. On the other hand, with shorter signals, there are fewer Transformers and correspondingly fewer temporal segments for these Transformers (with shared parameters) to learn from.

Although the proposed models are found to be comparable or better than SOTA results, there might be a practical limit in the actual discriminative eye-related information underlying EEG signals. An alternative hypothesis is that more sophisticated solutions, different approaches or further pre-processing or data cleaning might be required, as suggested in very recent work [19, 27, 32, 36]. To get insights into this, other approaches may be explored, such as graph neural networks, self-supervised learning, or exploiting additional past temporal information, as suggested by Kastrati et al. [17].

## Conclusion

We have presented novel deep learning models for fixation event prediction and gaze location estimation from EEG signals. We have considered a variety of models combining spatio-temporal and global-vs-local representations. Two of the proposed models compare favourably to existing SOTA models. One of them encodes spatial information among channels at a temporally local level first (via multiple Transformers), and then at a more global temporal level (via LSTMs). This model is more effective than a model which does not consider so explicitly the spatial relationships, but also more sensitive to the length of the considered signal, in particular with the reduced EEG channel set. This echoes the “no-free-lunch” theorem, and the importance of carefully tailoring the models not only to particular problems but also to specific data conditions. More generally, accurately predicting gaze location from EEG remains an interesting and challenging problem.

## Appendix. Details of the architectures

### A.1 Fixation event prediction

Our Model A was used by simply applying softmax at the output, getting fixation probability scores $\textbf{p} =(p_\text {fix},p_\text {no fix})$. The usual $\hat{y} = \arg \max _i p_i$ was applied for class prediction. The loss function for training was binary cross-entropy.

### A.2 Gaze location prediction

The loss function used in training is the mean squared error (Eq. 8).

#### CNN part

Both the CNN-1D used in Model A and the CNN-2D used in Model B rely on the same architecture with minor changes. A building convolutional block (Table 3) is sequentially repeated four times with increasing number of filters *k*: 16, 32, 64, and 128, respectively. The filter and pooling sizes are shown in Table 3.

**Table 3:** The building convolutional block parameterised by (*k*, *m*, *n*, *q*, *r*)

#### Transf-Enc

The Transformer encoder used in Model B consists of 6 layers, each of 4 heads and havin ReLU as activation function. The dropout layers have dropout rate $p=0.1$. The batch normalisation layer was applied *before* each FeedForward and multi-head attention blocks, not *later* [45], so that the range of the outputs of these blocks is larger and the resulting embeddings are more expressive. A final activation ReLU layer at the output, before being fed to the LSTM, was found to be important for the performance.

#### LSTM

This is a single-layer LSTM with hidden vector of dimension 768.

#### FC

The fully connected part consists of two layers with 384 and 2 units, respectively. ReLU activation function is used after the first layer.

#### Weight initialisation

Based on the observation of the lack of proper loss evolution in some particular training runs, weight initialisation was decided to He uniform for the CNN and the Transformer, and Xavier for the LSTM in Model B.

#### Model hyperparameters

Training details for both models are given in Table 4. AdamW refers to the decoupling the weight decay from the optimisation steps [29]. The patience is the number of epochs used during the early stopping monitoring on the validation loss.

**Table 4:** Training details in the proposed models

	Model A	Model B
Batch size	64	50
Learning rate	$8\cdot 10^{-4}$	$10^{-4}$
Max epochs	20	50
Optimizer	AdamW	
Patience	5	10

## Footnotes

1 Actually, GNNs and Transformers are related [15].

2 https://www.unicorn-bi.com/product/unicorn-hybrid-black/
